# Canine cutaneous lymphoma of large granular lymphocytes non-B, non-T natural killer immunophenotype with cardiac, gastric and hepatic involvement

**DOI:** 10.29374/2527-2179.bjvm000526

**Published:** 2026-07-24

**Authors:** Felipe Noleto de Paiva, Gabriela Oliveira Pereira, Vinícius Alves Furtado, Thiago Souza Costa, Bartolomeu Benedito Neves dos Santos, Vivian de Assunção Nogueira Carvalho, Julio Israel Fernandes

**Affiliations:** 1 Universidade Federal Rural do Rio de Janeiro, Seropédica, RJ, Brazil.; 2 Centro Universitário Goyazes, Trindade, GO, Brazil.

**Keywords:** cancer, immunohistochemistry, lymphoma, natural killer cell, câncer, imuno-histoquímica, linfoma, célula natural killer

## Abstract

Cases of natural killer (NK)-cell large granular lymphocyte lymphoma are extremely rare, accounting for approximately 0.8% to 2.56% of lymphomas in dogs. A 5-year-old male Rottweiler presented with multiple cutaneous nodules and progressive weight loss, with a clinical course of less than 2 months. Following inconclusive cytology, an incisional biopsy was performed for histopathological and immunohistochemical analyses. Histopathological examination revealed a neoplastic proliferation of round cells infiltrating the deep dermis and subcutaneous tissue. The microscopic features, combined with the immunostaining profile—negative for CD3 and CD79a and positive for CD18 and granzyme—indicated a diagnosis of large granular lymphocyte lymphoma with a non-B/non-T immunophenotype of NK-cell origin. The patient underwent chemotherapy with lomustine and completed three treatment sessions before being euthanized, with a survival time of 93 days. Necropsy revealed involvement of the lymph nodes, heart, stomach, liver, and skin. Histopathological and immunohistochemical findings confirmed the antemortem diagnosis at all sites. To the best of our knowledge, this is the first report of cutaneous, cardiac, gastric, and hepatic involvement associated with non-B, non-T large granular lymphocyte lymphoma in dogs.

## Resumo

Casos de linfoma de grandes linfócitos granulares de células natural killer (NK) são extremamente raros, representando aproximadamente 0,8% a 2,56% dos linfomas em cães. Um cão da raça Rottweiler, macho e com 5 anos de idade, apresentou múltiplos nódulos cutâneos e perda de peso progressiva, com um curso clínico inferior a dois meses. Após citologia inconclusiva, realizou-se biópsia incisional para análises histopatológica e imuno-histoquímica. O exame histopatológico revelou uma proliferação neoplásica de células arredondadas infiltrando a derme profunda e o tecido subcutâneo. As características microscópicas, combinadas com o perfil de imunomarcação — negativo para CD3 e CD79a e positivo para CD18 e granzima —, indicaram o diagnóstico de linfoma de grandes linfócitos granulares com imunofenótipo não-B/não-T de origem em células NK. O paciente foi submetido à quimioterapia com lomustina e completou três sessões de tratamento antes de ser submetido à eutanásia, apresentando um tempo de sobrevida de 93 dias. A necropsia revelou comprometimento de linfonodos, coração, estômago, fígado e pele. Os achados histopatológicos e imuno-histoquímicos confirmaram o diagnóstico *antemortem* em todos os locais. Até onde sabemos, este é o primeiro relato de acometimento cutâneo, cardíaco, gástrico e hepático associado ao linfoma de linfócitos granulares grandes não B, não T em cães.

## Introduction

Large granular lymphocyte (LGL) lymphomas are an extremely rare histological subtype of lymphoma (McDonough & Moore, 2000). They may originate from cytotoxic T lymphocytes or, less frequently, from natural killer (NK) cells (Hattori et al., 2018; Kotb et al., 2021; Turinelli et al., 2005). NK cells remain poorly studied in dogs, mainly because of the lack of specific markers for this cell type. Therefore, they are characterized as non-B and non-T, or “null-cell,” LGLs because they are negative for the standard T- and B-lymphocyte markers (Cora et al., 2017; Grudzien et al., 2021).

The actual incidence of NK-cell LGL lymphoma is unknown because of the challenges associated with its diagnosis. However, studies have reported an incidence ranging from 0.8% to 2.56% of all canine lymphomas (Cora et al., 2017; Jark et al., 2020; Kotb et al., 2021; Ponce et al., 2010). Here, we report a case of non-B, non-T NK-cell LGL lymphoma that initially presented with cutaneous lesions and subsequently involved the heart, stomach, and liver. To the best of our knowledge, no similar case has been reported in the literature.

## Case report

**Figure 1:**
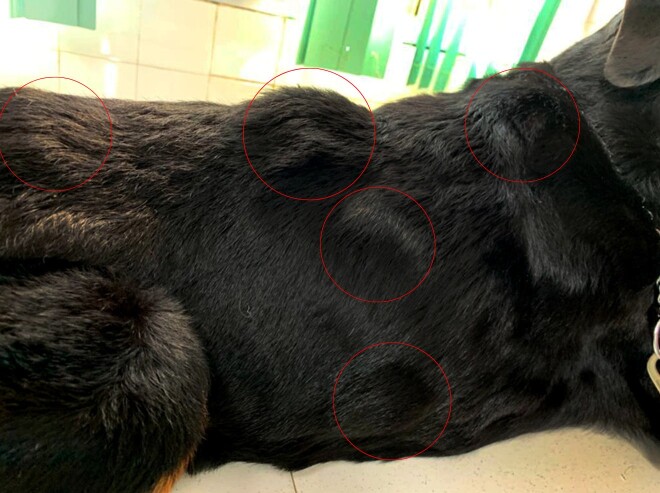
Canine patient presenting multiple cutaneous masses, predominantly in the dorsal region (circles).

A 5-year-old male Rottweiler was referred with multiple skin nodules and progressive weight loss of less than 2 months’ duration. Physical examination revealed vital parameters within the normal range for the species, and no abnormalities were detected on palpation of the lymph nodes. The nodules were predominantly located in the dorsal region, presenting as plaque-like lesions with increased volume. They were adherent, nonulcerated, soft to firm in consistency, and ranged from 3.0 to 6.0 cm in their largest dimension (Figure 1).

At the initial consultation, cytological examination was performed but yielded inconclusive results. A complete blood count and serum biochemical profile (creatinine, urea, alanine aminotransferase, alkaline phosphatase, albumin, and total protein) were performed, along with abdominal ultrasonography, thoracic radiography, and echocardiography, all of which revealed no abnormalities. For diagnostic purposes, an incisional biopsy was performed, and three skin fragments were collected from different lesions, focusing on the largest lesions. Histopathological and immunohistochemical analyses were subsequently performed.

**Figure 2:**
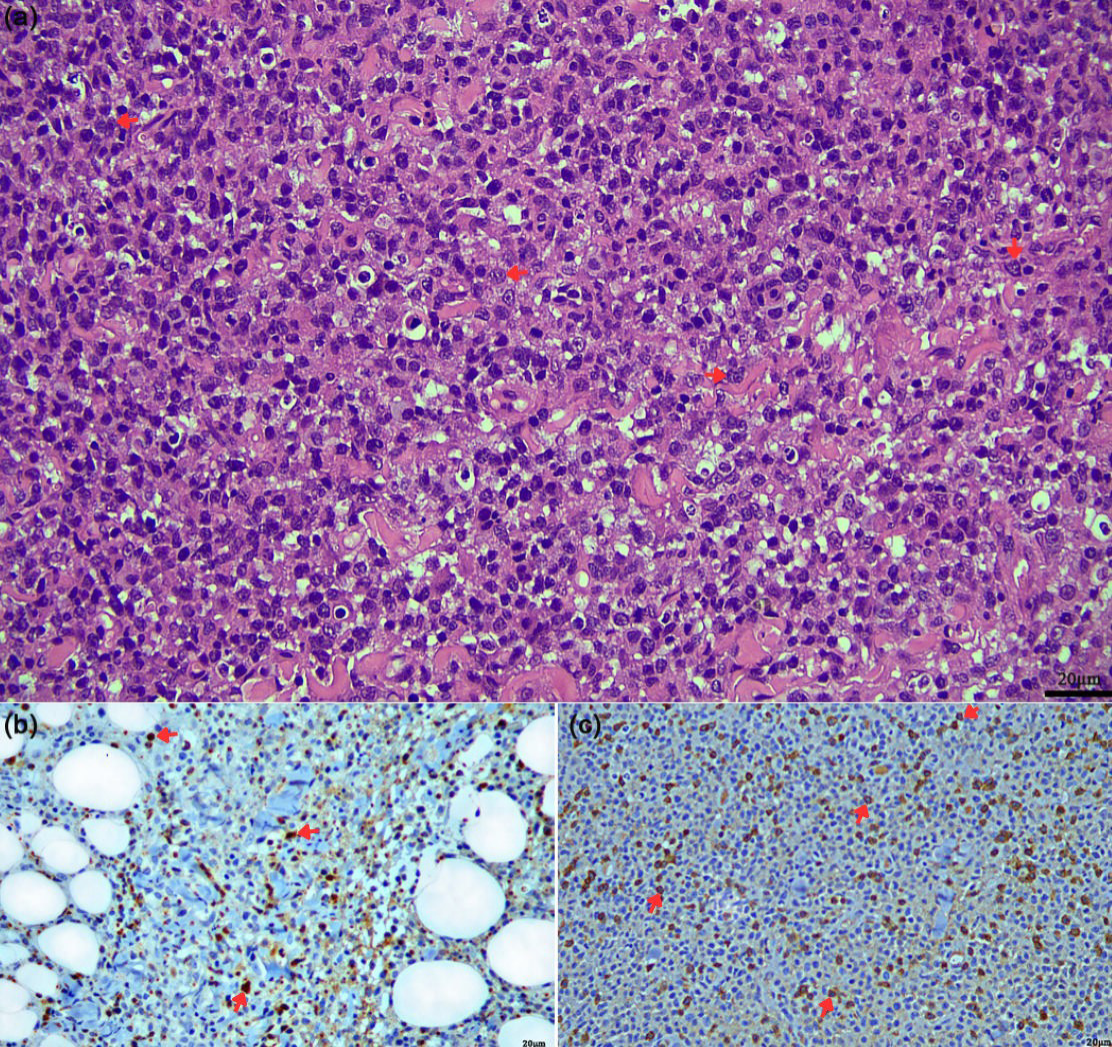
Histology of non-B and non-T large granular lymphocyte lymphoma in a dog. (A) Proliferation of round cells in the deep dermis with atypical mitotic figures (arrows), HE, 40× objective; (B) Positive immunohistochemical staining for CD18 (arrows), IHC, 40× objective; (C) Positive immunohistochemical staining for granzyme (arrows), IHC, 40× objective.

Histopathological examination revealed a neoplastic proliferation of round cells infiltrating the deep dermis and subcutaneous tissue. The neoplastic cells had indistinct cell borders, eosinophilic and slightly vacuolated cytoplasm, elliptical nuclei with condensed to finely granular chromatin, and one or more prominent nucleoli. Thirty mitotic figures were observed in 10 high-power fields (40× objective; Figure 2A). Immunohistochemical analysis showed no immunostaining for CD3, CD79a, MUM1, E-cadherin, c-Kit, or Melanoma Cocktail antigens. In contrast, diffuse membranous to cytoplasmic immunostaining for CD18 (Figure 2B) and cytoplasmic immunostaining for granzyme in approximately 60% of the neoplastic cells (Figure 2C) were observed. The immunohistochemical profile, together with the morphological features, supported a diagnosis of non-B, non-T LGL lymphoma of NK-cell origin.

The patient returned 10 days later to begin treatment, showing worsening of its general clinical condition and progression of the skin lesions, with multiple nodules disseminated throughout the trunk. The nodules were adherent, some were ulcerated, and all had a firm consistency. The largest lesion measured 15.0 × 12.0 cm. Chemotherapy with lomustine was initiated at an oral dose of 70 mg/m^2^ every 21 days. The dog underwent the first three chemotherapy sessions, maintaining stable disease, and the dose was progressively increased. Before the fourth session, the dog’s condition worsened markedly, with hyporexia, weight loss, anemia, and a considerable increase in lesion size. Euthanasia was performed, resulting in a survival time of 93 days from the initial consultation.

**Figure 3:**
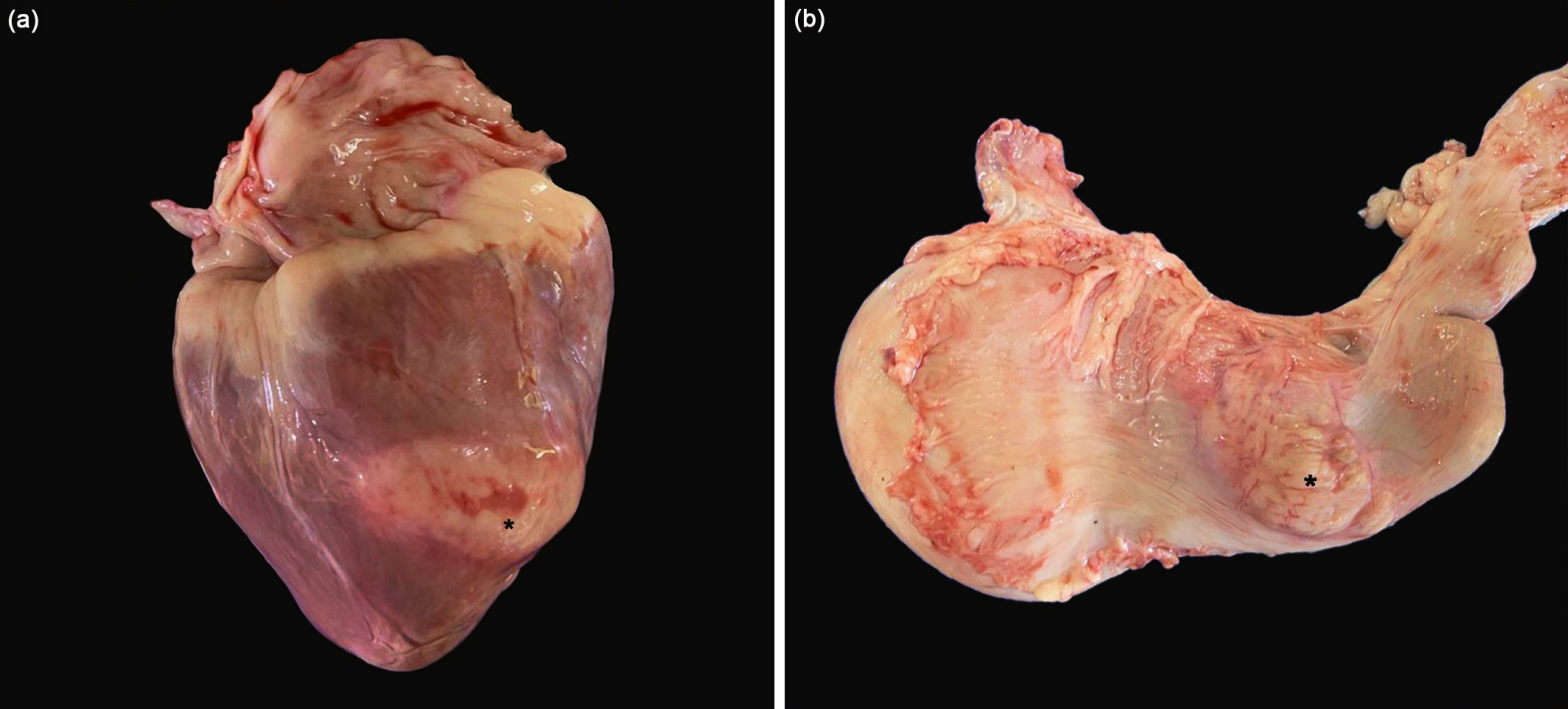
Necropsy findings in a dog with non-B and non-T large granular lymphocyte lymphoma. (A) Epicardial surface of the left ventricle showing an elliptical, raised, yellow mass (asterisk); (B) Raised, yellow mass on the serosa of the gastric antrum (asterisk).

Necropsy revealed moderate enlargement of the subscapular, retropharyngeal, and submandibular lymph nodes, as well as marked enlargement of the mesenteric lymph nodes. Cardiac examination revealed a white, raised, firm mass measuring 4.0 × 2.0 cm beneath the epicardial surface of the left ventricle (Figure 3A), extending into the underlying myocardium. The serosal surface of the gastric antrum also contained an irregular, white, raised, firm mass measuring 4.0 × 3.0 cm (Figure 3B). The liver was markedly enlarged and contained multiple small yellowish foci on the surface of all hepatic lobes. Microscopic examination of these tissues revealed a cellular proliferation similar to that observed in the skin biopsy specimens. Histopathological and immunohistochemical findings were consistent with those of the cutaneous lesions, confirming the diagnosis of LGL lymphoma with involvement of the skin, heart, stomach, liver, and lymph nodes.

## Discussion

Dogs are considered an excellent comparative model for the study of human lymphoma due to similarities at the molecular, pathological, and epidemiological levels (Foltz et al., 2016; Seelig et al., 2016). In humans, the first reports of lymphoproliferative disorders associated with LGLs date back to 1970, prompting extensive studies. These disorders were divided into two cell lineages, originating from either cytotoxic T lymphocytes or NK lymphocytes (McDonough & Moore, 2000). The clinical course can be acute or chronic (Turinelli et al., 2005), although LGL lymphoma often exhibits aggressive behavior with a high mortality rate (Kotb et al., 2021). The disease has also been reported in other species, including cats, horses, rats, ferrets, and birds (Turinelli et al., 2005). However, in dogs, LGL lymphoma remains poorly understood and studied (McDonough & Moore, 2000).

In veterinary medicine, as in human medicine, this tumor exhibits aggressive behavior, characterized by rapid progression (Kotb et al., 2021), with clinical signs including weight loss, lethargy, diarrhea, and frequent hepatosplenic involvement (Turinelli et al., 2005). In the present case, an extremely aggressive clinical course was observed, with similar clinical signs and rapid progression from the initial diagnosis, when no other organs were affected, to euthanasia, when the disease had become disseminated.

Most cases reported in dogs involve the lymph nodes (Cora et al., 2017; Varvil et al., 2022), with a single report of LGL lymphoma involving the kidneys (Kotb et al., 2021) and another involving the skin and stomach (Hattori et al., 2018); however, both were of the T-cell immunophenotype (Hattori et al., 2018; Kotb et al., 2021). There is also a report of a case involving the skin and bladder, although the cell of origin was not determined (Adachi et al., 2022). In cats, LGL lymphoma occurs more commonly with gastrointestinal involvement and is also more frequently associated with the T-cell immunophenotype (Hattori et al., 2018; Turinelli et al., 2005). No previous reports describing clinical manifestations similar to those observed in the present case were found in the literature.

The diagnosis of LGL lymphoma can be challenging because of its origin from cytotoxic T or NK cells, which are mainly located in extranodal tissues (McDonough & Moore, 2000; Turinelli et al., 2005). Histopathological evaluation mainly reveals large cell size and the presence of cytoplasmic azurophilic granules. In immunohistochemical evaluation, the following profiles are observed: CD3−, CD79a−, CD8−, CD4− in non-B, non-T lymphomas, and CD3+, CD79a−, CD8+, CD4− in T-cell cytotoxic lymphomas (Hattori et al., 2018; Turinelli et al., 2005).

Although NK lymphocytes remain poorly described and understood in dogs, they are often characterized by a non-B, non-T immunophenotype, granzyme positivity, and a large granular lymphocyte morphology (Foltz et al., 2016; Grøndahl-Rosado et al., 2015; Kim et al., 2019; Michael et al., 2013). In the present case, the diagnosis was confirmed based on the histopathological findings together with the CD18+, CD3−, CD79a−, and granzyme+ immunohistochemical profile.

The recommended treatment is similar to that for other types of lymphoma, with chemotherapy being the treatment of choice. However, there is no established chemotherapy protocol for LGL lymphoma (Turinelli et al., 2005). In the case described by Hattori et al. (2018), therapeutic success was achieved by combining surgical intervention with the Madison Wisconsin chemotherapy protocol, resulting in a survival time of 508 days. In the case reported by Adachi et al. (2022), nimustine chemotherapy was administered because of the initial cutaneous manifestation, following the recommendation for the use of a nitrosourea-based agent (Risbon et al., 2006; Williams et al., 2006), resulting in a survival time of 356 days. In the present report, lomustine was used as a single agent because of its indication for cutaneous lymphoma, resulting in stable disease for approximately 3 months and an overall survival time of 93 days. Due to the scarcity of reports, no survival estimates are available for this type of neoplasm. Nevertheless, a poor prognosis is expected because of its aggressive behavior.

## Conclusions

In conclusion, little is known about canine LGL lymphomas, especially those originating from non-B, non-T NK lymphocytes, because of the lack of specific markers and the limited knowledge of this cell type. However, the few published reports have consistently described an aggressive disease with a poor response to treatment. To the best of our knowledge, this is the first report of cutaneous, cardiac, gastric, and hepatic involvement associated with a non-B, non-T NK-cell immunophenotype LGL lymphoma in dogs.
